# Observational study of Reflux-related findings at distal esophagus, gastric pouch, and anastomotic site one and three years after one-anastomosis gastric bypass: exploring the diagnostic accuracy of endoscopy compared to biopsy

**DOI:** 10.1186/s12876-025-04510-w

**Published:** 2025-12-18

**Authors:** Mohamed Hany, Eman Sheta, Walid El Ansari

**Affiliations:** 1 Department of Surgery, Medical Research Institute, Alexandria University, Alexandria, Egypt; 2 Madina Women’s Hospital, Alexandria University, Alexandria, Egypt; 3 Department of Pathology, Faculty of Medicine, Alexandria University, Alexandria, Egypt; 4 College of Medicine, Ajman University, Ajman, United Arab Emirates

**Keywords:** Reflux, OAGB, Endoscopy, Diagnostic accuracy, Biopsy, GERD

## Abstract

**Background:**

We aimed to compare upper endoscopy (UE) with histopathology (biopsy) for detecting reflux-related findings at 1 and 3 years following one-anastomosis gastric bypass (OAGB).

**Methods:**

This retrospective analysis included 150 OAGB patients. UE (macroscopic) and biopsy (microscopic) findings were collected from the distal esophagus, gastric pouch, and anastomotic site. Five diagnostic indices—sensitivity, specificity, positive predictive value, negative predictive value, and area under the receiver operating characteristic curve—were calculated to assess UE relative to biopsy.

**Results:**

The mean age was 34.7 ± 11.4 years; 75.3% were female. At the distal esophagus, normal UE findings increased from 75.7% at year 1 to 92.7% at year 3 (odds ratio [OR] = 0.32, *p* = 0.005). Gastric pouch abnormalities rose from 57.6% to 73.4% (OR = 2.03, *p* = 0.007). Anastomotic site abnormalities increased from 16.0% to 21.7% (OR = 1.46, *p* = 0.226). Biopsy findings for the distal esophagus showed a nonsignificant increase in abnormalities (OR = 1.54, *p* = 0.103), whereas gastric pouch abnormalities rose significantly (OR = 3.45, *p* < 0.001). Diagnostic accuracy varied by anatomical region and follow-up interval. UE reliably detected but poorly excluded distal esophageal disease. At the gastric pouch, UE could both identify and rule out disease with high accuracy. At the anastomotic site, UE findings closely mirrored biopsy results, making it a potential substitute when biopsy is unavailable.

**Conclusions:**

Longer-term follow-up is needed post-OAGB. While UE effectively identifies distal esophageal disease, it may yield false negatives. By contrast, UE at the gastric pouch and anastomotic site reliably detects or excludes disease, offering a viable alternative to biopsy. Research of UE’s diagnostic accuracy at various time points in post-OAGB populations is required.

## Key points

1. At the distal esophagus, the incidence of normal upper endoscopy (UE) findings improved significantly over time (75.7% to 92.7%), while the gastric pouch (57.6% to 73.4%) and anastomotic site (16.0% to 21.7%) showed increasing abnormalities.

2. Diagnostic accuracy varied by anatomical region and follow-up interval: UE reliably detected distal esophageal disease but struggled to exclude it, whereas it accurately identified and ruled out disease at the gastric pouch.

3. At the anastomotic site, UE findings closely mirrored biopsy results, indicating that UE can be a viable substitute for biopsy when the latter is unavailable.

## Background

Globally, in 2022, one in every eight individuals was living with obesity [1]. These patients frequently experience obesity-associated medical complications, such as metabolic syndrome, type 2 diabetes mellitus, premature osteoarthritis, obstructive sleep apnea, and cardiovascular diseases, which altogether contribute to a heightened risk of mortality [2–4]. Metabolic and bariatric surgery (MBS) significantly improves weight and associated medical problems [5–7], and one-anastomosis gastric bypass (OAGB) has emerged as the third most common MBS procedure worldwide [8–10].

OAGB is effective in treating obesity, producing optimal weight loss, and reducing associated medical problems [11–13]. It is acknowledged by the American Society for Metabolic and Bariatric Surgery (ASMBS) [14] and increasingly recognized by the International Federation for the Surgery and Other Therapies for Obesity (IFSO) [15, 16]. OAGB nevertheless remains controversial due to concerns regarding postoperative reflux. For instance, IFSO has expressed apprehension about the potentially higher risk of reflux following OAGB, and a study reported reflux in up to 70% of patients and macro-/microscopic gastroesophagitis in 58% [16, 17]. Conversely, other findings indicate that the incidence of new-onset reflux at three years was higher, or at least similar, after sleeve gastrectomy than after OAGB [5, 18]. Given such inconsistencies, investigators advocate upper endoscopy (UE) and/or biopsy to confirm the diagnosis [19, 20].

Although UE and biopsy are frequently employed to detect reflux and related conditions after MBS, several knowledge gaps persist. Despite OAGB’s rising popularity and routine reliance on these diagnostic tools, no study has comprehensively compared their concordance or discordance in identifying reflux-related abnormalities. Previous investigations that used UE and biopsy concurrently after OAGB [19, 21, 22] had limitations, including short follow-up durations (6 months to 2 years), small sample sizes, and a lack of formal evaluations of UE’s diagnostic accuracy relative to biopsy at multiple postoperative time points [17, 20, 22–24].

Addressing these gaps is essential for clinicians and stakeholders involved in OAGB care. Therefore, the current study aims to clarify the controversies surrounding post-OAGB reflux by comparing UE with biopsy at one and three years after surgery, thereby providing more definitive guidance on diagnostic accuracy and the evolution of reflux-related findings over time.

## Methods

### Study design and ethics

We conducted a retrospective cohort study using prospectively collected data from patients who underwent OAGB at Alexandria Medical Research Institute, Alexandria, Egypt, between November 2017 and June 2018. Data were collected at baseline, one-year follow-up (during 2019), and three-year follow-up (2021) after OAGB. The study was approved by the Ethics Committee at our institution. Written informed consent was obtained from all patients who agreed to participate in the study. The study was reported in line with STROBE guidelines for observational research (Supplementary Table 1).

### Aim of the study

The current study aimed to appraise the macroscopic (UE) and microscopic (histopathological) changes at year 1 and year 3 after OAGB. The objectives comprised four interlinked questions at three anatomical levels (distal esophagus, gastric pouch, and anastomotic site): (1) What are the UE findings?; (2) What are the biopsy findings?; (3) What is the diagnostic accuracy of UE compared to biopsy (‘reference standard’), using five indices [sensitivity, specificity, positive/negative predictive values, area under the receiver operating characteristic (ROC) curve (AUC)]?; and, (4) What are the implications of the identified diagnostic accuracy for the use of UE in the detection of probable histopathological reflux-related abnormalities?

### Inclusion and exclusion criteria

Eligible participants were adults who underwent primary OAGB with a preoperative body mass index (BMI) > 40 kg/m², or > 35 kg/m² with obesity-related conditions. All patients had completed a standardized preoperative workup, including upper endoscopy.

Exclusion criteria included patients with any previous MBS, preoperative GERD symptoms, or preoperative endoscopic findings suggestive of reflux (e.g., esophagitis, hiatal hernia ≥ 5 cm, or Barrett’s esophagus). This was undertaken to ensure that only patients without preexisting reflux pathology were assessed for de novo postoperative changes [25].

### Preoperative evaluation of patients

A comprehensive assessment was undertaken by a multidisciplinary team (MDT) comprising a metabolic bariatric surgeon, nutritionist, psychiatrist, and endocrinologist. In line with others, appropriate nutrition, surgical, and psychological plans were discussed with each patient for optimal preparation [26, 27]. Laboratory, nutritional tests, ultrasound (for identifying calculous cholecystitis), and UE, to identify hiatal hernia (HH) and GERD using the LA classification [28, 29] were routinely performed for all patients. Helicobacter pylori was tested using a rapid urease test on endoscopic gastric biopsies and was eradicated preoperatively when present. In line with others, patients were required to stop smoking and alcohol consumption at least 4–6 weeks before surgery [30]. In addition, patients who were on regular proton pump inhibitors (PPI) or H2 blockers were asked to stop their medication two weeks before the UE [31–33]. Preoperative UE was routinely performed to identify and exclude patients with GERD, esophagitis, or Barrett’s esophagus (BE). However, these findings were not longitudinally analyzed, as the primary focus of this study was on postoperative macroscopic and microscopic changes.

### Surgical technique: OAGB

All procedures were undertaken by a single experienced metabolic bariatric surgeon (Table 1). The surgeon stood between the patient’s legs in a modified lithotomy position, using five ports, and began by dissecting the lesser omentum below the crow’s foot to create a window for the stapler. A long gastric pouch was created using the same linear stapler and reloaded over a 40-Fr bougie. The first reload was fired transversely below the level of incisura angularis followed by vertical reloads toward the angle of His with dissection of the angle of His and all posterior gastric adhesions. After measuring the whole bowel length, a 200 cm biliopancreatic limb length was attempted for BMI ≥ 50 kg/m^2^, and a 150 cm biliopancreatic limb length for BMI < 50 kg/m^2^ while keeping a common limb length of at least 300 cm. Blue reloads were used for gastrojejunostomy construction. Stapling defects were closed with continuous sutures using 3/0 V-Loc 180 sutures (Covidien, USA). The gastric pouch was measured by tape to ensure ≥ 15 cm length above the gastrojejunostomy.

**Table 1:** Key features of one-anastomosis gastric bypass surgical technique

Feature	Description
Bougie size	40 Fr
Width of pouch	2.5–3.0 cm
First stapler fire (lower pouch limit)	Below the level of incisura angularis
Last stapler fire	1.0–1.5 cm lateral to esophagogastric junction
His angle dissection	Yes
Length of pouch	15–20 cm above gastrojejunostomy
Capacity of pouch	40–60 mL
Counting the whole bowel length	Yes
Biliopancreatic limb length	200 cm for BMI score of ≥ 50 kg/m^2^ 150 cm for BMI score of < 50 kg/m^2^ Common limb length of at least 300 cm
Width of gastroenterostomy	3–4 cm
Reinforcement	Oversewing invaginating seromuscular sutures
Hiatal hernia repair	Yes, if preoperatively diagnosed
Methylene blue test	Yes

*BMI* body mass index

Staple lines were reinforced with an invaginating series using the same barbed sutures. Closure of mesenteric defects was not needed. Crural repair for hiatal hernia using 2/0 V-Loc nonabsorbable sutures (Covidien, USA) was attempted in all cases with preoperatively diagnosed HH. An intraoperative methylene blue leak test was routine, and a tube drain was routinely placed in the left subphrenic space.

### Upper gastrointestinal endoscopy protocol

Preoperatively, all patients underwent UE under conscious sedation after fasting for 6–8 h, performed by the same experienced endoscopist using a high-definition gastroscope. This baseline assessment focused on the distal esophagus, stomach, and esophagogastric junction to exclude patients with large hiatal hernia, BE, or pre-existing GERD (including LA-graded esophagitis).

During postoperative follow-up, UE was scheduled at 12 and 36 months, or earlier if patients developed reflux-related or dyspeptic symptoms. At each examination, the distal esophagus, gastric pouch, and anastomotic site were assessed with systematic biopsy collection. GERD was not diagnosed by random biopsies; instead, biopsies were reserved for suspected BE or mucosal pathology, as GERD outcomes were outside the scope of this study. Patients with preoperative GERD or LA-graded esophagitis were excluded to ensure assessment of de novo mucosal changes.

HH was diagnosed when an upward displacement of the esophagogastric junction of >2 cm above the diaphragmatic hiatus (axial length of >2 cm) was seen on UE with widening of the diaphragmatic crural impression as seen on retroversion view of the scope (Hill grade IV) [34]. The axial length of the HH was measured using the hash marks on the scope during withdrawal from the endoscope. Patients with HH were excluded from the study. The presence or absence of bile in the esophagus and gastric pouch was noted.

Barrett’s esophagus was identified during endoscopy by salmon-pink mucosa extending above the gastroesophageal junction, with histologic confirmation of specialized intestinal metaplasia. During the 2017–2021 study period, our protocol mandated systematic distal esophageal biopsies, including at the squamocolumnar junction, in line with prevailing research standards of that time [17, 35, 36]. Current guidelines now distinguish BE from an irregular z-line (< 1 cm) and advise against routine biopsy of the latter [37], but this nuance was not uniformly adopted when our data were collected.

A standardized template was completed at the end of each UE, documenting findings at each anatomical level as normal or with mucosal changes (e.g., gastritis, hyperemia, ulceration), recognizing that many represent common findings not necessarily requiring intervention. Hiatal hernia was diagnosed when the esophagogastric junction was displaced >2 cm above the diaphragmatic hiatus, with widening of the crural impression (Hill grade IV). Incompetent cardia was defined as failure of closure of the gastroesophageal junction on retroflexion. Gastric pouch abnormalities (erosive and non-erosive gastritis) were classified in reference to the Sydney system for gastritis [38], while anastomotic site changes (hyperemia, ulcer) followed descriptions used in post-bypass endoscopic frameworks [26, 39]. This systematic approach ensured consistency across all examinations and time points, as all procedures were performed by a single experienced endoscopist. PPIs or H2 blockers were discontinued two weeks before the UE in patients using these medications [30].

### Biopsy (histopathology) protocol

Endoscopic biopsies from the three sites (esophagus, gastric pouch, anastomosis site) were fixed immediately in 10% formalin. After 24 h, fixed tissues were processed into paraffin blocks. Five-micron-thick sections were cut and mounted on glass slides to be stained by hematoxylin and eosin (H&E) stain. Slides were examined under a light microscope by an experienced pathologist for any pathological changes and were blinded to the UE findings.

In esophageal biopsies, cases were reported as normal if mucosal fragments were covered by stratified squamous epithelium with no inflammation, metaplasia, or dysplasia. Biopsies were not employed to diagnose GERD, in line with guideline recommendations, and were not taken from areas of active erosive esophagitis. Their purpose was limited to excluding BE or alternative diagnoses such as eosinophilic esophagitis. If inflammatory cells were seen within the esophageal epithelial lining associated with basal cell hyperplasia with or without lamina propria inflammation, esophagitis was reported. To avoid sampling errors, BE was diagnosed with intestinal metaplasia with evident goblet cells in correlation with the UE biopsy site and UE findings.

For gastric pouch biopsies, chronic gastritis was reported if features of chronicity were seen, e.g., glandular architectural changes, lamina propria inflammation, or fibrosis. The activity was diagnosed by neutrophils in glands (cryptitis or crypt abscess) or lamina propria. H. pylori was suggested when gastritis was associated with lymphoid follicle formation with or without visible organism in H&E staining, further confirmed using anti-H. pylori immunohistochemical stain.

In biopsies from the anastomotic site, jejunal fragments were assessed for pathologic changes. Chronic inflammation was diagnosed when features of chronicity were seen as villous shortening, crypt distortion, and lamina propria inflammation. Ulceration was reported if the surface epithelium was sloughed, which was associated with the presence of granulation tissue, fibrin debris, or degenerative changes.

### Postoperative care

Enoxaparin prophylaxis against venous thrombosis was started 12 h before surgery and continued for ≥ 3 weeks after surgery. Patients were discharged once they tolerated a liquid diet with prescription for supplements. All patients received routine proton pump inhibitor (PPI) therapy for 12 months postoperatively, as part of standardized institutional care. UE was conducted for all participants after 1 and 3 years to detect any GERD, HH, marginal ulcers (MUs), and de novo H pylori infection. HH was diagnosed postoperatively as an upward displacement of the esophagogastric junction above the diaphragmatic hiatus with an axial length of >2 cm [34].

### Statistical analysis

Descriptive statistics included means and standard deviations (M ± SD) for continuous data, and frequencies and percentages for categorical data. Diagnostic accuracy of UE compared to biopsy was assessed using sensitivity, specificity, positive predictive value (PPV), negative predictive value (NPV), and ROC AUC. 95% confidence intervals for these metrics provided estimates of the precision. In line with others, ROC AUC was interpreted as: 0.5 = no discrimination; >0.5 to < 0.7, poor; 0.7 to < 0.8, acceptable; 0.8 to < 0.9, excellent; and AUC ≥ 0.9 as outstanding discrimination. To estimate the changes in endoscopic and biopsy findings between year 1 and year 3 post-OAGB surgery, a Generalized Estimating Equations (GEE) approach was employed. Statistical analyses were performed using R software version 4.2 and ‘pROC’ package [40].

## Results

**Fig. 1:**
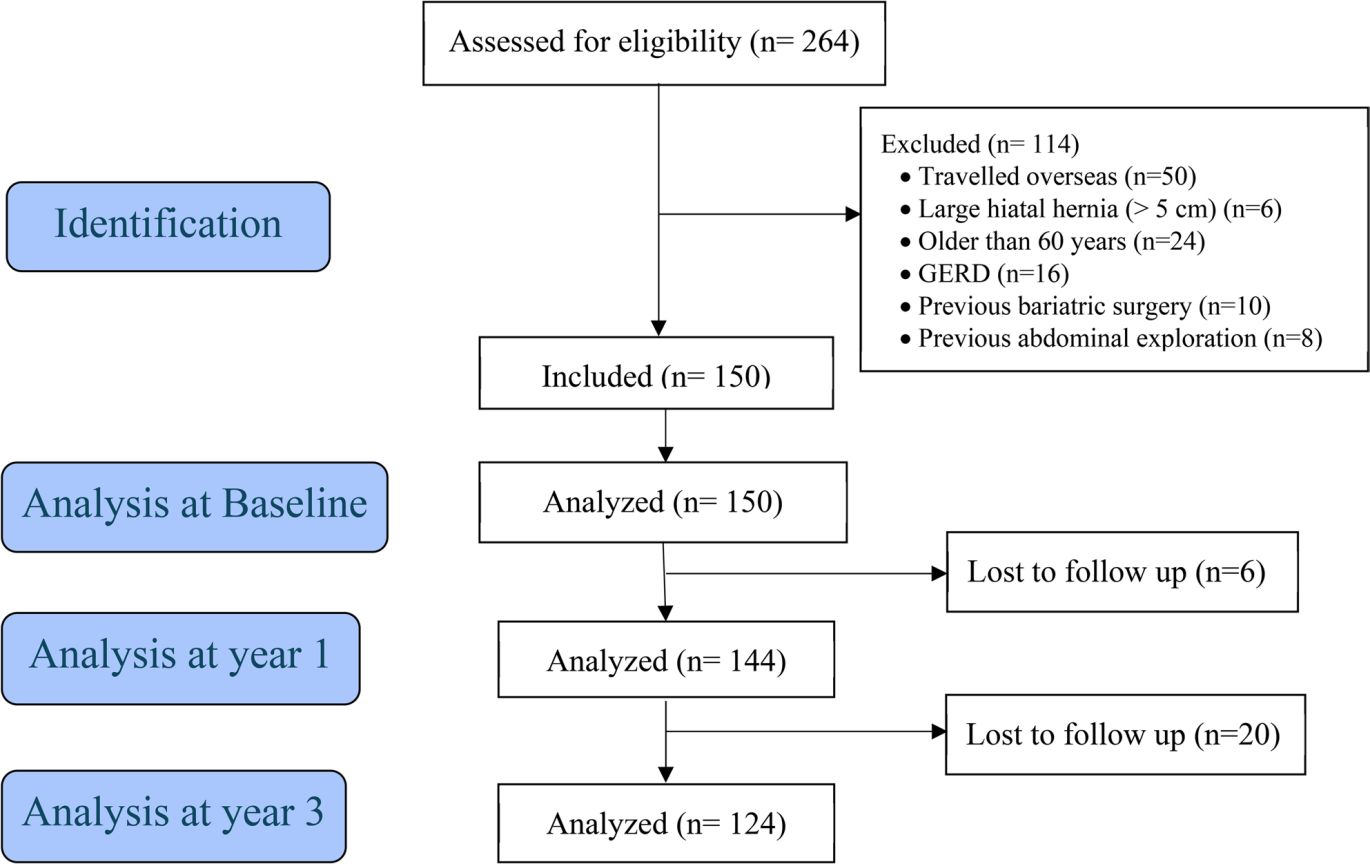
Participant flow chart

A total of 264 consecutive patients undertook OAGB during the study period. One hundred fourteen patients were excluded as they did not meet the inclusion criteria. Of these, 50 patients had returned home overseas. The remaining 150 patients consented and were included in the study. At year 1, six patients were lost to follow-up, and at year three, 20 were lost to follow-up. Figure 1 shows the participant flow chart.

### Baseline characteristics

The mean age was 34.7 ± 11.4 years, 75.3% were female, and 12.7% of patients reported smoking. The mean pre-op BMI was 43.8 ± 3.2 kg/m^2^ (Table 2). At years 1 and 3 follow-up, the percent total weight loss (TWL%) was 42.2 ± 0.3% and 42.7 ± 0.8% (*p* = 0.95), and the percent excess weight loss (EWL%) was 99.7 ± 9.6% and 100.7 ± 10.5% (*p* = 0.92) (Table 3).

**Table 2:** Preoperative characteristics of the sample (*N* = 150)

Variable	Value *n* (%)
Age (years)	
M ± SD	34.7 ± 11.4
Median (Range)	32.5 (18–60)
Sex	
Female	113 (75.3)
Male	37 (24.7)
Smoking	
Yes	19 (12.7)
No	131 (87.3)
BMI (kg/m^2^)	
M ± SD	43.8 ± 3.2
Median (Range)	43.42 (36.0–54.1.)

Cell values represent number frequency unless otherwise stated, M ± SD, mean ± standard deviation, *BMI* body mass index

**Table 3:** Selected characteristics of the sample at two time points

Variable	Year 1 (*N* = 144)	Year 3 (*N* = 124)
Weight (kg)	67.3 ± 5.6	66.9 ± 6.0
BMI (kg/m^2^)	25.3 ± 1.8	25.2 ± 2.0
TWL%	42.2 ± 0.3	42.7 ± 0.8
EWL%	99.7 ± 9.6	100.7 ± 10.5

Cell values represent mean ± standard deviation (M ± SD), *BMI* body mass index, *TWL%* Total weight lost percent, *EWL%* excess weight lost percent

### Upper endoscopy and biopsy (histopathological) findings at two time points

In terms of UE findings, at the distal esophagus, there was a significant improvement in the proportion of normal findings from 75.7% at year 1 to 92.7% at year 3, with the odds of having an abnormal finding at year 3 significantly reduced to an OR of 0.32 (95% CI: 0.15, 0.72; *p* = 0.005). Conversely, at the gastric pouch, the odds of having an abnormal finding increased, with an OR of 2.03 (95% CI: 1.21, 3.40; *p* = 0.007), reflecting a deterioration from 57.6% abnormal findings at year 1 to 73.4% at year 3. The anastomotic site showed a non-significant increase in abnormalities, from 16.0% at year 1 to 21.7% at year 3 (OR: 1.46, 95% CI: 0.79, 2.71; *p* = 0.226), indicating a trend towards worsening, though not statistically significant (Table 4).

**Table 4:** Endoscopy and biopsy findings of the sample at two time points after OAGB

Finding	Year 1 (*n* = 144)	Year 3 (*n* = 124)	OR* (95% CI)	*p*
Endoscopy				
Distal Esophagus				
Normal	116(75.7)	115(92.7)		
Abnormal	28(24.3)	9(7.3)		
Hiatus Hernia	3(2.1)	4(3.2)	0.32 (0.15, 0.72)	*0.005*
Incompetent Cardia	24(16.7)	4(3.2)		
Mucosal Abnormality	4(2.8)	4(3.2)		
Bile	6(4.2)	5(4.0)		
Gastric pouch				
Normal	61(42.4)	33(26.6)		
Abnormal	83(57.6)	91(73.4)		
Non-erosive gastritis	59(41.0)	60(48.4)	2.03 (1.21, 3.40)	*0.007*
Erosive gastritis	24(16.7)	31(25.0)		
Anastomotic site				
Normal	121(84.0)	97(78.2)		
Abnormal	23(16.0)	27(21.7)		
Ulcer	3(2.1)	4(3.2)	1.46 (0.79, 2.71)	0.226
Mucosal hyperemia	20(13.9)	23(18.5)		
Biopsy				
Distal esophagus				
Normal	53(36.8)	34(27.4)		
Abnormal	91(63.2)	90(72.6)		
Chronic Inflammation	91(63.2)	90(72.6)	1.54 (0.92, 2.59)	0.103
Barrett’s esophagus	0(0)	1(0.8)		
Gastric Pouch				
Normal	77(53.5)	31(21.5)		
Abnormal	67(46.5)	93(75)		
Chronic Gastritis	66(45.8)	91(73.4)		
Active (erosive)	3(2.1)	4(3.2)	3.45 (2.05, 5.81)	*< 0.001*
Non-active	66(45.8)	91(73.4)		
H. Pylori	6(4.2)	8(6.5)		
Anastomotic Site				
Normal	121(84.0)	97(78.2)		
Abnormal	23(16.0)	27(21.7)		
Chronic Inflammation	20(13.9)	23(18.5)	1.46 (0.79, 2.71)	0.226
Ulcer	3(2.1)	4(3.2)		

Cell values represent frequency (%), *OR* Odds ratio, CI Confidence Interval'

* odds of abnormal finding observed at year 3 compared to year 1

**Fig. 2:**
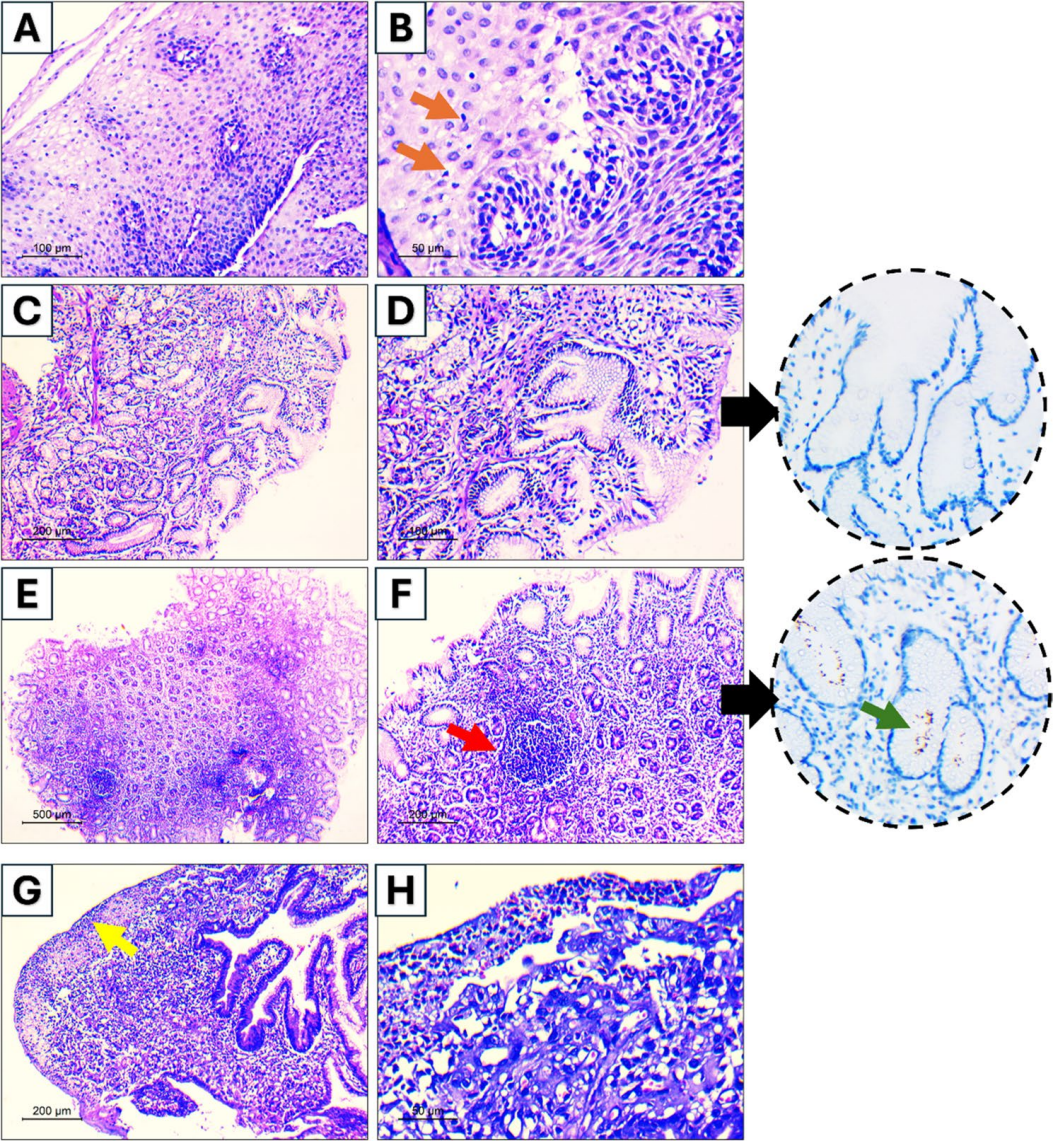
Histopathologic assessment of some included cases. **A** Esophageal biopsy showing acanthosis and basal hyperplasia; **B** Higher power view showing few inflammatory cells (orange arrows) indicating esophagitis; **C**, **D** Gastric pouch biopsy showing chronic inflammation of lamina propria with architectural changes, inset shows negative Helicobacter pylori immunostain; **E**, **F** Another gastric pouch biopsy exhibiting chronic inflammation of lamina propria with lymphoid follicle formation (red arrow), inset shows positive brown stained organisms (green arrow) in glands lumens in Helicobacter immunostain; **G** Anastomotic site biopsy showing ulceration of surface mucosa (yellow arrow); **H** Higher power shows granulation tissue

Table 4 also shows that for the biopsy findings, at the distal esophagus, although the proportion of abnormal findings increased from 63.2% at year 1 to 72.6% at year 3, with odds ratio (OR) of 1.54 (95% CI: 0.92, 2.59; *p* = 0.103, not statistically significant). The gastric pouch showed significant deterioration, with odds of abnormal findings increasing substantially to 3.45 (95% CI: 2.05, 5.81; *p* < 0.001), reflecting an increase in abnormalities from 46.5% to 75%. This included a significant rise in chronic gastritis. At the anastomotic site, there was a non-significant increase in the rate of mucosal changes at the anastomotic site, most of which represented common findings of limited clinical impact (OR: 1.46, 95% CI: 0.79, 2.71; *p* = 0.226). Figure 2 depicts the histopathologic assessment of some included cases.

### Diagnostic accuracy: endoscopy compared to biopsy

Table 5 presents an exploratory concordance analysis between UE and biopsy at the two time points. In terms of sensitivity and specificity, at year 1, UE exhibited the highest accuracy indices at the anastomotic site, together with high sensitivity at the gastric pouch level, and high specificity at the distal esophagus level. Generally, this configuration did not change much at year 3.

**Table 5:** Diagnostic accuracy of endoscopy compared to biopsy at two time points after OAGB

Endoscopy		Biopsy			Accuracy Indices				
		Abnormal	Normal		Sensitivity	Specificity	PPV	NPV	ROC AUC
Year 1									
Distal Oesophagus	Abnormal	28	0		30.8 (20.7–40.0)	100 (100–100)	100 (100–100)	45.7 (36.6–54.1)	0.65 (0.61–0.70)
	Normal	63	53						
Gastric Pouch	Abnormal	66	17		98.5 (92.4–100)	77.9 (66.3–86.1)	79.5 (69.5–87.5)	98.4 (92.1–100)	0.88 (0.83–0.93)
	Normal	1	60						
Anastomotic Site	Abnormal	23	0		100 (100–100)	100 (100–100)	100 (100–100)	100 (100–100)	1 (1–1)
	Normal	0	121						
Year 3									
Distal Esophagus	Abnormal	8	1		8.9 (4.1–16.6)	97.1 (84.2–100)	88.9 (33.3–100)	28.7 (20.9–37.3)	0.53 (0.49–0.57)
	Normal	82	33						
Gastric Pouch	Abnormal	91	0		97.8 (93.0–100.0)	100 (100–100)	100 (100–100)	93.9 (78.8–100)	0.99 (0.97–0.99)
	Normal	2	31						
Anastomotic Site	Abnormal	27	0		100 (100–100)	100 (100–100)	100 (100–100)	100 (100–100)	1 (1–1)
	Normal	0	97						

Cell values represent n; values for accuracy indices represented as mean (95% Confidence interval), *PPV* positive predictive value, *NPV* negative predictive value, *ROC* receiver operating characteristic curve, *AUC* area under the curve

**Fig. 3:**
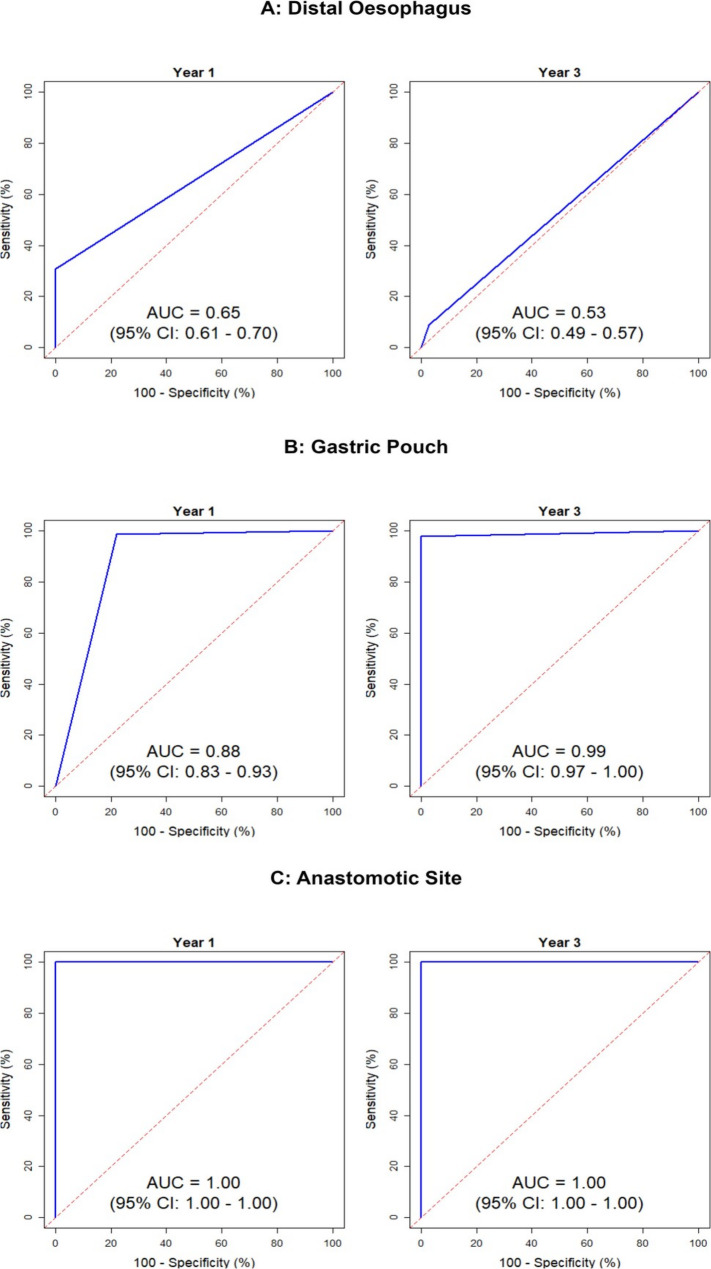
ROC curves of endoscopy vs. biopsy at two time points after OAGB. **A** Distal esophagus; **B** gastric pouch; and **C** anastomotic site

**Fig. 4:**
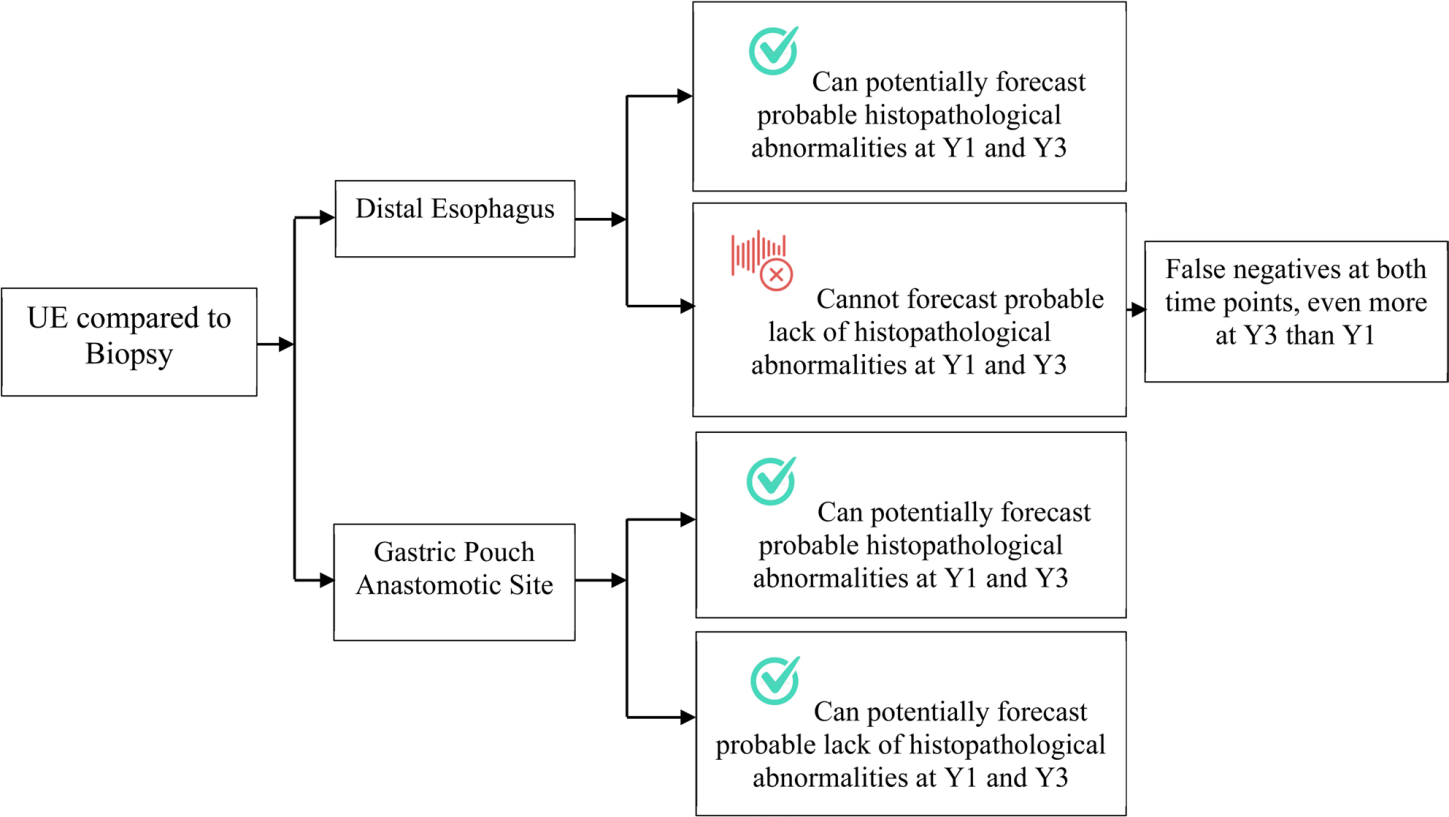
Utility of upper endoscopy in forecasting biopsy outcomes by anatomic level and time points. *UE* upper endoscopy; *Y* year

Figure 3 shows the ROC AUC. At both time points, the area under the curve was very large for the anastomotic site, quite large at the gastric pouch, and much less for the distal esophagus. Table 6; Fig. 4 summarize the diagnostic accuracy indices of UE compared to biopsy and their clinical implications.

**Table 6:** Diagnostic accuracy of UE compared to biopsy: indices by anatomic level and time points and their implications

Anatomic Level/Accuracy Indices	Year 1		Year 3	
	UE indices	Comment	UE indices	Comment
Distal Esophagus				
Specificity, PPV	High specificity + PPV (both 100%)	Effective for forecasting probable abnormalities	Small decline but still very high specificity (97.1%) + high PPV (88.9%)	Still effective for forecasting probable abnormalities
Sensitivity, NPV	Very low sensitivity (30.8%) + NPV (45.7%)	Much less reliable for forecasting probable lack of abnormalities, many true abnormalities may be missed	Notable decline in sensitivity to 8.9% + NPV to 28.7%	Further limiting UE’s utility in forecasting probable lack of abnormalities
ROC AUC	0.65	Poor overall discriminatory ability	Further decreased to 0.53 with 95%CI crossing 0.5	No discriminatory ability
Gastric Pouch				
Specificity, PPV	Fairly good specificity (77.9%) + PPV (79.5%)	Good for forecasting probable abnormalities	Specificity much improved to 100%, perfect PPV (100%)	Good for forecasting probable abnormalities
Sensitivity, NPV	High sensitivity (98.5%) + NPV (98.4%)	Effective for forecasting probable lack of abnormalities	High sensitivity (97.8%) + NPV (93.9%)	Effective for forecasting probable lack of abnormalities
ROC AUC	0.88	Good overall discriminatory ability	0.99 (almost perfect)	Good overall discriminatory ability
Anastomotic Site				
Specificity, PPV, sensitivity, NPV, ROC AUC	Good diagnostic metrics at Y1 and Y3, with 100% sensitivity, specificity, PPV, and NPV, as well as ROC AUC of 1. Such performance means it is ideal for both forecasting probable abnormalities and lack of abnormalities, maintaining its discriminatory ability across the study period			

*UE* upper endoscopy, *PPV* positive predictive value, *NPV* negative predictive value, *ROC AUC* area under the receiver operating characteristic (ROC) curve (AUC), *CI* confidence interval, *Y* year

The concordance between endoscopic and histological abnormalities was assessed in an exploratory analysis. This was not intended as a diagnostic standard for GERD, as random biopsies are not recognized diagnostic tests for reflux disease.

## Discussion

This retrospective analysis used prospectively collected data from patients who underwent OAGB to evaluate the diagnostic accuracy of endoscopy (macroscopic) compared with biopsy (microscopic) findings. Five accuracy indices were applied across a large cohort of post-OAGB patients at two points. To the best of our knowledge, this is the first study to undertake such a detailed assessment, a task essential for building robust evidence to resolve existing controversies surrounding post-OAGB reflux.

Technical aspects of OAGB may influence postoperative reflux. The loop gastrojejunostomy configuration can predispose to biliary reflux, whereas pouch length and width, the angle of the gastrojejunostomy, and the orientation of the anastomosis are theorized to mitigate bile exposure. Standardization of these parameters may be essential to reducing reflux incidence; however, no previous research has conducted an in-depth comparison of UE and biopsy as diagnostic tools to detect post-OAGB reflux-related abnormalities. Although OAGB is a safe and effective procedure frequently performed worldwide with documented metabolic benefits, there is a risk of reflux and its theoretical carcinogenic potential [17]. Diagnosing reflux remains challenging, and no single diagnostic method is superior [41].

The one-year (short-term) interval was selected to capture any early reflux manifestations after OAGB, aligning with other studies that assessed UE and histopathological changes over 6- to 24-month periods [19, 20]. The three-year (mid-term) time point was chosen to: (a) explore the influence of time on gastroesophageal reflux disease (GERD), if present, based on macroscopic (UE) or microscopic (biopsy) evidence; (b) bridge existing knowledge gaps, given that previous post-OAGB studies in Brazil and Poland assessed UE, histopathological changes, or BE only up to two years [19, 20], and (c) address calls for further medium- and long-term investigations of reflux following MBS [10, 17, 19, 42, 43]. Importantly, all patients with pre-existing reflux symptoms or endoscopic findings suggestive of GERD were excluded preoperatively. This enabled a focused evaluation of de novo postoperative mucosal changes and their progression over time. The 3-year follow-up allowed us to document a gradual increase in gastritis and mucosal abnormalities, contributing novel longitudinal data to the limited literature on late mucosal outcomes after OAGB.

The diagnostic accuracy of UE compared with biopsy for reflux-related abnormalities varied notably across anatomical sites and time points. UE displayed good specificity and PPV at the distal esophagus at year 1, with only a slight decrease at year 3. In the gastric pouch, UE demonstrated fairly good specificity and PPV at year 1, which improved substantially at year 3. In contrast, UE exhibited good specificity and PPV at both time points at the anastomotic site. Below, we discuss these findings in further detail.

Our study observed incompetent cardia via UE in 16.7% of patients at year 1, decreasing to 3.2% at year 3. The UE appearance of the cardia can identify individuals with pathological gastroesophageal reflux, and the presence of a defective lower esophageal sphincter is an independent predictor of abnormal esophageal acid exposure. These findings underscore that the competence of the gastroesophageal junction is an essential determinant of reflux susceptibility [44].

As for non-erosive or erosive gastritis detected by UE, the rates increased from 57.6% at year 1 to 73.4% at year 3. This aligns with the 61.5% overall (non-erosive or erosive) gastritis rate reported at two years post-OAGB [20]; the data suggest a time-dependent rise in gastritis prevalence: 57.6% at year 1 (our study), 61.5% at year 2 (Braga et al. [20]), and 73.4% at year 3 (our study). The progressive increase in both non-erosive (from 41% to 48%) and erosive gastritis (from 16% to 25%) between the first and third postoperative years may reflect cumulative mucosal exposure to biliary refluxate—a well-recognized phenomenon in OAGB due to the absence of a pyloric barrier. A systematic review reported bile reflux rates ranging from 7.8% to 55.5%, depending on technique, follow-up duration, and diagnostic criteria [45]. Shenouda et al. further demonstrated that elevated bilirubin concentrations (>20 mg/dL) in gastric aspirates were significantly associated with erosive inflammation, providing biochemical evidence of bile-mediated mucosal damage [23]. This aligns with our findings and is consistent with other longitudinal studies demonstrating increased bile reflux over time after OAGB [46].

The progressive increase in gastric mucosal abnormalities observed in our cohort raises concerns about potential long-term consequences of chronic bile exposure. While early surgical literature linked bile reflux after Billroth II reconstruction to an increased risk of gastric cancer, more recent bariatric surgery studies provide a nuanced perspective. A matched cohort study demonstrated a significant reduction in esophageal and gastric cancer incidence following bariatric surgery compared to non-surgical controls [47]. However, a Korean nationwide cohort study found that gastric cancer, although rare, can still occur after bariatric surgery and is often diagnosed at a more advanced stage [48, 49]. While our study was not designed to evaluate malignancy, our findings of progressive mucosal injury underscore the importance of long-term surveillance. We recommend adherence to the IFSO guidelines for surveillance endoscopy at 1, 3, and 5 years, followed by 10-year intervals, to monitor for premalignant changes [43].

In the present study, MU incidence was 2.1% at year 1, slightly increasing to 3.2% at year 3. These findings are consistent with systematic reviews reporting MU rates of 0.6–4% in large cohorts [50, 51], and with a recent pooled random-effects analysis indicating that 3% of patients experience MU after primary OAGB [18]. Interestingly, although our MU rate rose modestly between year 1 and year 3, the meta-analysis above found that MU frequency declined over longer follow-up, dropping from 3% within the first 5 years to 2% beyond 5 years [18]. The reasons underlying this discrepancy remain unclear.

Regardless of bile reflux (BR), several other risk factors have been recognized for MU, including increased acid production in an oversized gastric pouch [52]. Some investigators propose that the presence of bile within the anastomotic region in OAGB may have a protective buffering effect against the ulcerogenic action of acid [53]. Although most studies hold that MU risk factors after OAGB largely mirror those observed after RYGB (Roux-en-Y Gastric Bypass)—such as smoking, untreated Helicobacter pylori, chronic NSAID (non-steroidal anti-inflammatory drugs) use, and the absence of postoperative proton pump inhibitors [12, 54–56], others suggest that certain procedure-specific details, including a longer pouch, biliopancreatic irritation, and anastomotic tension, may facilitate MU formation after OAGB [57].

The present study noted BE rates of 0% at year 1 and 0.8% at year 3. These findings align with a recent pooled fixed-effects analysis of 15 studies, which reported a 1% incidence of BE after OAGB [18]. Interestingly, bile in the distal esophagus among patients with GERD does not appear to correlate with BE as strongly as acid reflux does [58]. Moreover, a study employing impedance-24 h‐pH‐metry, high‐resolution manometry, and gastroscopy following OAGB demonstrated a decrease in acid reflux but an increase in non-acid reflux at mid-term follow-up of primary OAGB patients [59].

Pertaining to the diagnostic accuracy of UE compared with biopsy, our literature search did not reveal any prior studies suitable for direct comparison; to the best of our knowledge, no previous research has specifically examined this issue. Consequently, we agree that comparative data on reflux are lacking [17]. In our analysis, the diagnostic accuracy of UE relative to biopsy for reflux-related abnormalities varied notably according to both the anatomical site and the postoperative time point.

For the distal esophagus, UE demonstrated excellent year-1 capability for identifying (ruling in) histopathological abnormalities but limited capacity for excluding them (ruling out), with performance deteriorating by year 3 and potentially yielding numerous false negatives. Its ROC AUC also decreased from year 1 to year 3, signifying poor overall discriminative power. False negatives are clinically significant because they can lead to legal action and litigation, diminished public confidence, delayed diagnosis and treatment, and ultimately increased morbidity and mortality [60–62].

Although some post-OAGB reflux studies have employed UE and biopsy, none have explicitly evaluated diagnostic accuracy. For example, a study examining GERD-related histopathological and macroscopic findings two years following OAGB reported that while 48% of patients showed macroscopic esophagitis (Grades A and B), only 6% were confirmed to have reflux esophagitis on final pathological analysis [19].

At the gastric pouch level, the diagnostic performance of UE exhibited high sensitivity and NPV, with both measures increasing substantially from year 1 to year 3. Across both time points, UE was robust in ruling out histopathological abnormalities. Furthermore, its reasonably good specificity and PPV at year 1 improved to perfection by year 3, indicating that UE is also effective at ruling in histopathological abnormalities at this site.

A study that investigated GERD and esophagitis/gastritis after OAGB reported that 28.7% of patients had evidence of at least grade 1 remnant gastritis on endoscopy. In contrast, histopathological examination revealed mild or more significant gastric inflammation in 38.7% of patients [63]. Despite a significant difference between endoscopic and histopathological findings, no formal diagnostic accuracy assessment was performed. The authors proposed that the subjective interpretation of endoscopic appearances [63] might partly explain the lack of correlation.

Another prospective series examining bile reflux after OAGB via bile reflux scintigraphy and endoscopy found new abnormal UE findings in 39.5% of patients postoperatively, many of which were not readily apparent macroscopically. Notably, these postoperative UE findings were significantly associated with bile reflux scintigraphy results [46].

Together, our findings suggest that at the gastric pouch level, UE can be used confidently to identify and exclude histopathological disease at the time points evaluated in this study. Nonetheless, we concur with multiple authors who recommend incorporating gastric biopsy into UE follow-up to objectively and promptly detect advanced inflammation or malignant transformation [46]. This has practical implications, as it may support the selective use of biopsy in routine surveillance, particularly when endoscopic findings are normal at anatomical sites, with high diagnostic accuracy.

UE exhibited excellent diagnostic performance at the anastomotic site across all metrics evaluated, including sensitivity, specificity, PPV, NPV, and ROC AUC, all achieving 100%. These findings underscore its outstanding discriminative ability and suggest that UE can be used with exceptional confidence to detect and exclude histopathological abnormalities, even without a biopsy. Such high accuracy may reflect the nature of the abnormalities that typically occur at this site, most commonly marginal ulcers, which are relatively straightforward to identify microscopically. Additionally, some authors posit that the actual incidence of MU following various bariatric procedures may be underreported, given that current literature largely relies on studies where diagnostic testing was conducted on a selective basis, leading to considerable observation bias [20].

This study has limitations. It is a single-center study, which may limit the generalizability of the findings. Both PPV and NPV depend on the local prevalence of abnormalities, necessitating caution when applying these data to other populations. The ROC analyses at one anatomical level do not account for potential abnormalities at the other levels, and controlling for such effects was not feasible given the rarity of isolated abnormalities. Because our comparison between UE and biopsy focused on the presence (or absence) of any abnormality, it did not distinguish specific lesion types. PPI use was standardized during the first postoperative year; however, long-term adherence was not systematically recorded, and discontinuation of PPIs before UE was self-reported. These may have influenced the progression or resolution of mucosal abnormalities at follow-up and should be considered when interpreting the findings. Future studies should stratify patients based on actual PPI duration and adherence. The study did not employ validated reflux diagnostic standards such as LA grading or prolonged pH monitoring to report GERD incidence, restricting direct comparisons with reflux-focused studies. Future prospective designs should integrate these modalities to better define reflux outcomes alongside mucosal injury. Random biopsies are not a diagnostic standard for GERD, and their clinical utility is limited to conditions such as eosinophilic esophagitis, H. pylori infection, or gastric intestinal metaplasia. The current diagnostic accuracy analysis should therefore be interpreted as exploratory concordance between macroscopic and microscopic abnormalities, not as a GERD diagnostic tool. Current guidelines now distinguish BE from an irregular z-line (< 1 cm) and recommend against routine biopsy of the latter due to the risks of overdiagnosis and unnecessary surveillance [37]. This more nuanced practice was not uniformly adopted during the period when the current study was implemented but should be incorporated in future research for improved specificity and clinical relevance. More detailed scoring systems, such as the full Hill or AFS classifications for hiatal hernia, were not systematically employed in this study. Our approach prioritized internal consistency through standardized templates and a single experienced endoscopist, but future prospective studies should incorporate validated multi-parameter criteria to enhance reproducibility. The current study did not include a comparator arm stratified by preoperative PPI use or hiatal hernia status, nor a parallel bariatric cohort with a mechanistically distinct procedure (e.g., SG or RYGB). These limit the comparative insights as well as causal inference regarding whether observed mucosal changes are specific to OAGB or reflect broader postoperative phenomena. Future comparative designs are needed to address this. Longer-term outcomes would have been beneficial, as our follow-up was restricted to one and three years. Future investigations should incorporate additional diagnostic techniques (e.g., scintigraphy, manometry), longer follow-ups, and symptom assessments to further clarify the nature of acid versus biliary reflux. Finally, the lack of longitudinal analysis of preoperative endoscopic findings is a limitation. Future prospective studies incorporating baseline-to-follow-up comparisons would better clarify de novo mucosal changes.

Despite the limitations, the study has many strengths. It is the first to simultaneously appraise a large series of patients for macroscopic and microscopic changes at one and three years after OAGB, answering, four inter-linked questions at three anatomical levels (distal esophagus, gastric pouch, anastomotic site). More importantly, the study appraised the diagnostic accuracy of UE compared to biopsy among patients after OAGB and did so at more than one time point after the procedure, using five indices to meticulously dissect diagnostic performance by anatomic level and time point, thus providing clinical implications and recommendations for its utility and circumstances to detect or exclude histopathological disease or non-disease confidently.

## Conclusions

Upper endoscopy can reliably detect histopathological abnormalities at the distal esophagus, although it is less effective in ruling them out due to potential false negatives. Conversely, at the gastric and anastomotic sites, endoscopic findings demonstrated high diagnostic concordance with histopathology, supporting selective biopsy strategies during postoperative surveillance. In addition, a time-dependent increase in both non-erosive and erosive gastritis was observed, which may reflect chronic mucosal exposure to bile refluxate rather than infectious causes. These findings underscore the value of routine surveillance at 1, 3, and 5 years post-OAGB as recommended by IFSO, particularly in high-volume centers.

## Data Availability

Data are available upon reasonable request from the corresponding author and subject to agreement from the participating institution where the research was implemented.

## Abbreviations

ASMBS: American Society for Metabolic and Bariatric Surgery
AUC: Area under the curve
BE: Barrett's esophagus
BMI: Body mass index
BR: Bile reflux
CI: Confidence interval
GEE: Generalized estimating equations
GERD: Gastroesophageal reflux disease
H&E: Hematoxylin and eosin
H2 Blockers: Histamine type-2 receptor antagonists
HH: Hiatal hernia
IFSO: International Federation for the Surgery and Other Therapies for Obesity
LA: Los Angeles
M±SD: Mean ± standard deviation
MBS: Metabolic bariatric surgery
MDT: Multidisciplinary team
MUs: Marginal ulcers
NPV: Negative predictive value
NSAID: Non-steroidal anti-inflammatory drugs
OAGB: One-anastomosis gastric bypass
OR: Odds ratio
PPI: Proton pump inhibitors
PPV: Positive predictive value
PRISMA: Preferred reporting items for systematic reviews and meta-analyses
ROC: Receiver operating characteristic
RYGB: Roux-en-Y Gastric Bypass
UE: Upper endoscopy
